# Engineered Polyethylene Glycol-Coated Zinc Ferrite Nanoparticles as a Novel Magnetic Resonance Imaging Contrast Agent

**DOI:** 10.1021/acsbiomaterials.3c00255

**Published:** 2023-06-13

**Authors:** Shadab Dabagh, Somayeh Asadi Haris, Yavuz Nuri Ertas

**Affiliations:** † ERNAM—Nanotechnology Research and Application Center, Erciyes University, Kayseri 38039, Türkiye; ‡ Department of Biomedical Engineering, Erciyes University, Kayseri 38039, Türkiye

**Keywords:** magnetic nanoparticles, contrast agent, magnetic resonance imaging, relaxivity, ferrites

## Abstract

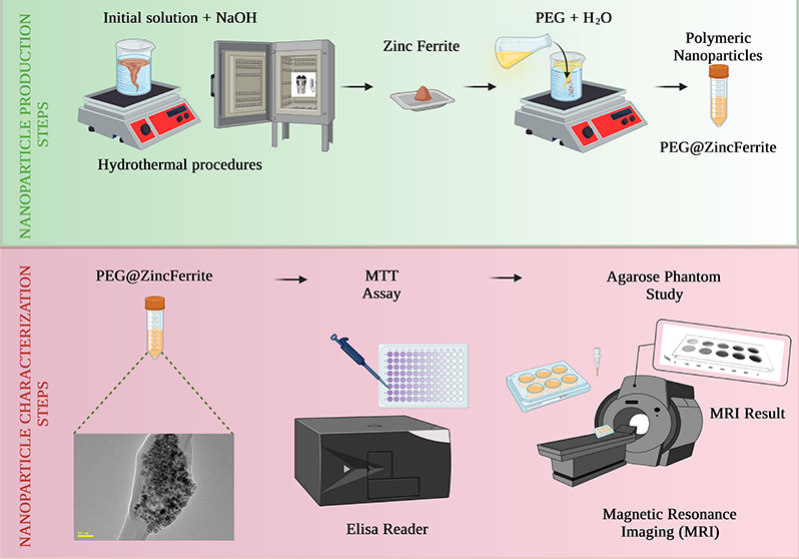

Polyethylene glycol (PEG) was utilized to functionalize the surface of zinc ferrite nanoparticles (NPs) synthesized by the hydrothermal process in order to prevent aggregation and improve the biocompatibility of the NPs for the proposed magnetic resonance imaging (MRI) agent. Various spectroscopy techniques were used to examine the NPs’ structure, size, morphology, and magnetic properties. The NPs had a cubic spinel structure with an average size of 8 nm. The formations of the spinel ferrite and the PEG coating band at the ranges of 300–600 and 800–2000 cm^–1^, respectively, were validated by Fourier-transform infrared spectroscopy. The NPs were spherical in shape, and energy-dispersive X-ray spectroscopy with mapping confirmed the presence of zinc, iron, and oxygen in the samples. The results of high-resolution transmission electron microscopy revealed an average size of 14 nm and increased stability after PEG coating. The decrease in zeta potential from −24.5 to −36.5 mV confirmed the PEG coating on the surface of the NPs. A high saturation magnetization of ∼50 emu/g, measured by vibration sample magnetometer, indicated the magnetic potential of NPs for biomedical applications. An MTT assay was used to examine the cytotoxicity and viability of human normal skin cells (HSF 1184) exposed to zinc ferrite and PEG@Zn ferrite NPs at various concentrations. After 24 h of treatment, negligible cytotoxicity of PEG-coated NPs was observed at high concentrations. Magnetic resonance imaging (MRI) suggested that PEG@Zn ferrite NPs are a unique and perfectly suited contrast agent for T_2_-weighted MRI and can successfully enhance the image contrast.

## Introduction

Recent progress in nanobiomaterial research has resulted in the discovery of magnetic nanoparticles (MNPs) with considerable promise for biomedical applications.^1−3^ It has proven possible to use magnetic nanoparticles in drug delivery,^4^ cell targeting via protein and small molecule binding, diagnostic applications,^5^ intracellular drug release,^6,7^ imaging,^8,9^ and combination treatments.^10,11^ Therefore, due to the unique physical features of MNPs that enable them to activate at the cellular and molecular levels of biological interactions, they have the potential to alter conventional clinical diagnosis and therapy^12,13^ and are commonly employed for medical diagnostics.^14^ Magnetic resonance imaging (MRI) is a painless and safe diagnostic procedure that employs a magnetic field and radio frequency to generate high-resolution images of the organs and structures of the body.^15,16^ MNPs also require a surface coating that is nontoxic and biocompatible and enables targeted distribution.^17^ As MRI contrast agents, paramagnetic or superparamagnetic metal ions are utilized that boost contrast sensitivity because these materials can cause changes in relaxation times (brighter/T_1_ and darker/T_2_).^18^ Metal ions that are paramagnetic or superparamagnetic can produce effective MRI contrast in the form of nearby spin water molecules (T_2_ or transverse relaxation rather than T_1_ or longitudinal relaxation).^19^ A number of MNP formulations are currently being developed for use in MRI; however, there exists a need to establish a mixed formulation for these specific demands. Superparamagnetic iron oxide nanoparticles have been widely investigated as MRI contrast agents, and various formulations have achieved clinical approval. Nevertheless, due to existing detection limits and a lack of specific identification, their widespread utility has yet to be realized.

Due to their low toxicity and high magnetic properties, iron oxide-based MNPs are particularly suitable for a number of applications. The formation of magnetic shells with different coating materials around particles^20,21^ and the modification of particle composition by introducing additional elements into their crystalline structure^22−24^ are the most common methods for producing NPs suitable for biomedical applications. Using one-pot decomposition, superparamagnetic manganese, cobalt, and zinc–iron-doped iron oxide NPs were produced. The prepared MNPs were investigated for their potential use as contrast agents in MRI and hyperthermia therapy agents. Cobalt substitution increased coercive fields at low temperatures, but zinc substitution significantly increased saturation magnetizations, and manganese had a smaller effect on overall magnetic properties. The transverse relaxation coefficients (r_2_) of the MNPs were significantly higher than 100 mM^–1^ s^–1^, indicating a substantial improvement over several commercially available T_2_-contrast agents based on pure iron oxide NPs.^25^ Elsewhere, the crystal structures, magnetic characteristics, and contrast abilities of manganese-doped magnetite NPs were reported, where, as the manganese doping level increased, the lattice distances and saturation magnetizations rose gradually. Manganese-doped magnetite NPs exhibited a saturation magnetization (*M_s_*) of 89.5 emu/g with an exceedingly strong T_2_ contrast effect with an r_2_ value of 904.4 mM^–1^ s^–1^ at 7.0 T. Compared to iron-oxide-based commercially available products, prepared NPs in the ratio of Mn/Fe (1:7) showed high T_2_ contrast ability and gave much greater signal sensitivity for imaging live subjects.^26^ An r_2_ relaxivity of 270 mM^–1^ s^–1^ and sensitive in vivo liver MRI in mice were achieved by encapsulating manganese-doped superparamagnetic iron oxide nanoclusters with the amphiphilic diblock copolymer methoxypoly(ethylene glycol)-*b*-poly(caprolactone) (mPEG-*b*-PCL).^27^ To limit quick absorption by the reticuloendothelial system and permit prolonged blood circulation, manganese ferrite nanoparticles were coated with a thick PEG shell, which also increased the stability of NPs in aqueous environments. The combination of biocompatibility, high T_2_ effect, and excellent r_2_/r_1_ values at low magnetic fields confer these NPs desirable properties as MRI contrast agents.^28^ Although recent literature reported many types of spinel-structured ferrites as MRI contrast agents, zinc is the most suitable candidate as a dopant since the Food and Drug Administration (FDA) recommends high reference daily values (DVs) for zinc and iron of 15 and 18 mg, which are significantly higher than the values for other potential dopants, such as manganese and cobalt at 2 mg.^16^ Chaudhary et al. optimized zinc-doped ferrite NPs (Zn_0.4_Fe_2.6_O_4_) with a diameter of 24 nm as MRI contrast agents and compared them to commercial ferrite NPs. A substantially higher enhancement in T_2_ (1.22-fold) and a slightly higher T_1_ (1.09-fold) contrast were reported compared to commercial ferrite nanoparticles. Zinc substitution not only enhanced MRI contrast properties but also significantly minimized the chances of iron overloading by iron cation substitution.^16^

Spinel ferrite NPs have superparamagnetic characteristics, although the majority of them display poor chemical stability, and as a consequence, their surfaces need to be modified. Furthermore, ferrite NPs have a high surface-to-volume ratio and are prone to aggregation. For effective MRI applications, ferrite NPs should be capped with biocompatible polymers to stabilize NP dispersions under physiological circumstances.^29^ As uncoated NPs face several obstacles, including cellular absorption, colloidal stability, and clearance by the reticuloendothelial system,^30^ conjugation with bioactive molecules is essential because it improves biocompatibility and blood circulation, enables optical detectability, and, most importantly, does not induce toxicity in the body.^31^ Therefore, covering the surface of nanomaterials with such polymers that are naturally abundant, hydrophilic, biocompatible, and biodegradable will boost the hydrophilicity and dispersibility of NPs, thereby enhancing their biocompatibility and biodegradability.^31,32^

PEG’s uncharged and hydrophilic properties, in addition to its low toxicity and immunogenicity, render PEG-coated NPs immune-invisible. These characteristics make PEG-coated NPs appealing for biological applications.^33,34^ PEG-diacid-functionalized MnFe_2_O_4_ NPs were synthesized using the solvothermal technique, and their cytotoxicity, MRI, and hyperthermia evaluations were performed, where a transverse relaxivity of 216 mM^–1^ s^–1^ was achieved. The PEG-diacid coating of the NPs offered colloidal stability appropriate for biological applications, and a cytotoxicity study on breast cancer and normal epithelial cell lines demonstrated that the prepared NPs were biocompatible but had a considerable toxic effect on breast cancer cells.^35^ Elsewhere, PEG coating was applied to functionalize La_1–*x*_Sr_*x*_MnO_3_ superparamagnetic NPs, and excellent colloidal stability, hemocompatibility, and biocompatibility were demonstrated. PEG-coated NPs exhibited a 3-fold r_2_ value compared to bare NPs.^36^

PEG has been the most studied synthetic hydrophilic polymer among other polymers. By limiting opsonization and providing steric hindrance, PEG has established itself as a good NP stabilizer.^37^ As a result, functionalization with PEG would slow down NP clearance by the reticuloendothelial system and increase their permeability and retention effect in vivo, thereby increasing the half-life of MNPs in circulation.^38,39^ Numerous parameters, including processing procedures, functionalization, and calcination/sintering, play a crucial role in ensuring that MNPs work ideally for their intended uses. These variables can have a substantial impact on the size and shape of the produced particles, making it necessary to pick the most suitable preparation procedure from the numerous synthesis approaches.^40^ Different types of ferrites have been synthesized using a variety of procedures, such as the microwave approach,^41^ solution combustion,^42^ hydrothermal decomposition,^43^ solid-state reaction,^44^ sol–gel,^45,46^ and coprecipitation.^47^ For the preparation of MNPs, hydrothermal synthesis^48,49^ is preferred due to its better yield, simplicity, low cost, and high degree of compositional control in terms of particle size and crystallinity.

Although zinc ferrite NPs with different types of surface coatings, such as silica,^50^ graphene oxide,^51^ chitosan,^52^ and PEG,^53^ were reported previously, saturation magnetization, cytotoxicity, and T_2_ contrast effect modifications need further investigation. To the best of our knowledge, there is insufficient information on the MRI contrast enhancement impact and the biocompatibility evaluation of PEG-coated zinc ferrite NPs. In this study, we report zinc ferrite NPs stabilized with a PEG coating (MW = 6000 g/mol), referred to as PEG@Zn ferrite NPs, with low toxicity and small crystallite size achieved via the hydrothermal synthesis method. The substitution of zinc to ferrite nanoparticles and coating with PEG resulted in biocompatible behavior and considerably controlled saturation magnetization (*M*_*s*_), hence improving MRI contrast while minimizing the risk of iron overloading through iron cation replacement and preventing the quick clearance of MNPs. This unique magnetic nanoparticle with improved magnetic characteristics and reduced adverse effects on the human body is a potential candidate for MRI applications.

## Materials and Methods

### PEG@Zn Ferrite NPs Synthesis

Hydrothermal synthesis was utilized to produce Zn ferrite NPs with a nominal composition of ZnFe_2_O_4_. The spinel structure of ZnFe_2_O_4_ is normal, with Zn^2+^ ions in the A-site and Fe^3+^ ions in the B-sites. The nanocrystalline ZnFe_2_O_4_ system always appears as a normal spinel with Zn^2+^ and Fe^3+^ ions distributed over the A- and B-sites; hence, the formula is (Zn_1−δ_^2+^Fe_δ_^3+^)[Zn_δ_^2+^Fe_2−δ_^3+^]O_4_^2–^, where δ is defined as the fraction of A-sites occupied by Fe^3+^ cations and is dependent on the technique of synthesis. δ for Zn ferrite prepared by the hydrothermal technique is zero considering a normal spinel.^54^ The distribution of Fe^3+^ into tetrahedral and Zn^2+^ into octahedral interstices is the main property of nanocrystalline ZnFe_2_O_4_ by the hydrothermal method. This cationic redistribution causes the formation of two magnetic sublattices, (A) and (B), which are responsible for the increased magnetization observed as compared to normal ZnFe_2_O_4_. As a result, nanosized ZnFe_2_O_4_ with a normal spinel structure has significantly higher magnetization.^55,56^ Iron(II) nitrate hexahydrate, zinc nitrate hexahydrate, sodium hydroxide (NaOH), and PEG (MW = 6000 g/mol) of 99% purity were acquired from Merck. All the nitrates were initially dissolved in distilled water. Then, drop by drop, NaOH solution was added to the stirring solution until the desired pH of 12 was reached. The precipitate was then deposited in an oven in a hydrothermal autoclave reactor for 10 h at 200 °C.^57^ The precipitates were washed many times with distilled water and 100% ethyl alcohol to remove unreacted products, then dried at 80 °C for 8 h, and calcined overnight at 500 °C for better crystallinity and magnetization. The chemical reaction is as follows:
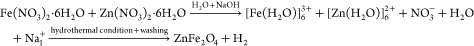


With an ultrasonic bath, 20 mg of ZnFe_2_O_4_ was mixed into 1 mL of deionized water to make the coated NPs. PEG–water was prepared by combining 75 mg of PEG with 1.5 mL of deionized water and stirring for 20 min. Slowly, the obtained solutions were added to the ZnFe_2_O_4_ MNPs. The finished solution was agitated at room temperature for more than 6 h, then collected, rinsed with deionized water, and dried in a vacuum oven at 50 °C overnight.

### Analytical Methods

Using a powder X-ray diffractometer (XRD, D8 Advanced), the purity and spinel structure of the produced samples at 2θ range from 10° to 90° with Cu Kα radiation (λ = 1.54065 Å) were measured. Fourier transform infrared (FTIR, Bruker Vector 22, Germany) spectroscopy was used to evaluate the integrity of the prepared NPs. After combining 1 mg of ferrite sample with 100 mg of potassium bromide (KBr), compressed pellets were used to collect the spectra in the range of 300–4000 cm^–1^. The hydrodynamic size (mean particle size) and zeta potential were validated using dynamic light scattering (DLS; Zeta sizer Nano ZS-90, Malvern, U.K.). A field emission scanning electron microscope (FESEM, JSM-6700F, JEOL, Japan) was used. FESEM snapshots were converted to 3D images using the ImageJ software. Utilizing an energy dispersive spectrum (EDS, Thermo Noran System 7), the exact metal ion composition of the MNPs was determined. A transmission electron microscope (TEM, Hitachi H7650, Japan) coupled with high-resolution TEM (HRTEM), operating at 100 kV, was used to image the NPs, which were prepared by dispersing in methanol before being drop-cast onto a carbon-coated copper grid and air-dried. A vibrating sample magnetometer (VSM, Lake Shore model 7307, U.S.A.) was used to obtain the magnetic properties of MNPs at room temperature.

### Cytotoxicity Studies

Human normal skin cells (HSF 1184) were cultured in high glucose DMEM media supplemented with 10% heat-inactivated fetal bovine serum (FBS, VWR, U.S.A.) and 1% penicillin–streptomycin (Sigma, St. Louis, U.S.A.). Cells were then maintained at 37 °C in a humid atmosphere containing 5% CO_2_ (v/v). All in vitro investigations were conducted during the exponential growth phase of the cells. The HSF 1184 cell line was cultured in flasks for 24 h, and its exponential growth phase was measured. Then, 200 μL of cell suspension containing 5 × 10^4^ cells per well was added to each well of a sterile 96-well microplate and incubated overnight at 37 °C, 5% CO_2_, and 98% humidity.^58^ The cells were then treated for 24 h with 50 μL of zinc ferrite and PEG-coated zinc ferrite nanoparticles (0–1000 μg/mL) Following this, 20 μL of MTT reagent in phosphate-buffered saline (PBS) containing 3-(4,5-dimethylthiazol-2-yl)-2,5-diphenyltetrazolium bromide was added to each well and incubated for 4 h. As negative controls, untreated cells, medium with 100% cell viability, and a blank were utilized. To dissolve the violet crystals produced from live cells, 200 μL of dimethyl sulfoxide solution was added to each well after the MTT solution was withdrawn. The absorbance was measured at 570 nm using an ELISA microtiter plate reader after 15 min. The experiments were carried out in triplicate, and the cell viability percentage was calculated using the following formula:^59^
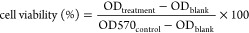


### Magnetic Resonance Imaging Using a Phantom

Using an agarose phantom study, the contrast ability of the samples was evaluated. To do this, various concentrations of PEG@Zn ferrite NPs were suspended in agarose gel (1%, w/v) and scanned using T_2_-weighted imaging protocols on a clinical 1.5T MR scanner (Avanto, Siemens, Germany). T_2_-weighted images were collected using the following parameters: 1.5T, fast spin–echo, repetition time TR = 2500 ms, echo time TE = 30–180 ms (increment of 6 ms), FOV = 16 cm^2^, resolution = 256 × 256 points, and slice thickness = 6 mm.

**Figure 1:**
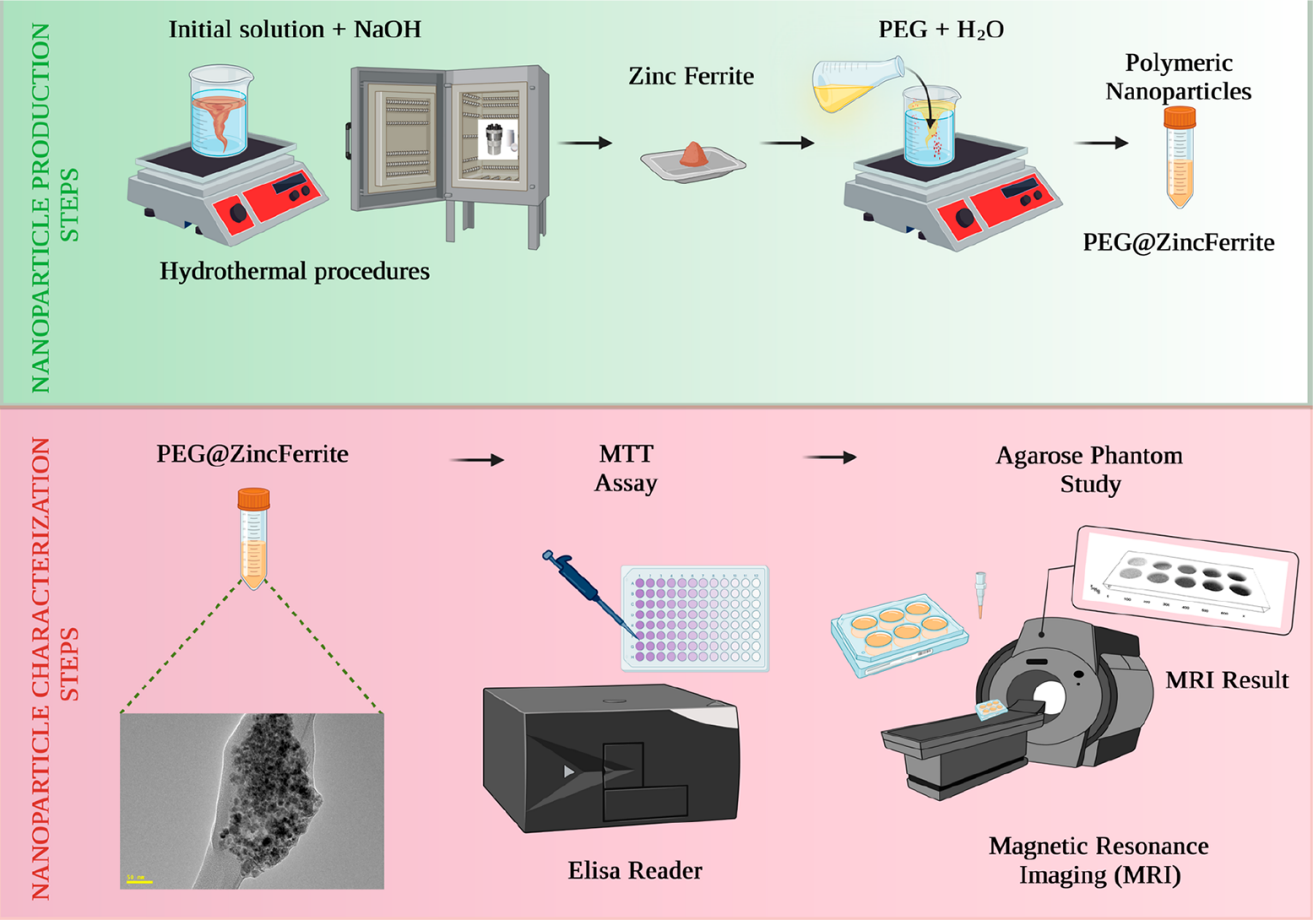
Schematics of experimental and analytical study of Zn ferrite and PEG@Zn ferrite NPs.

In a 96-well plate, various concentrations of Zn ferrite NPs and PEG@Zn ferrite NPs (in μg/mL) were suspended in 1% (w/v) agarose gel.^60^ The plate was put into a knee coil for MRI. By fitting a curve to plots of 1/T_2_ (in s^–1^) vs the total of the concentration (in μg/mL) of different concentrations of Zn ferrite NPs and PEG@Zn ferrite NPs, the values of r_2_ were determined. Figure 1 is a summary of the experimental and analytical study schematic diagram.

Ferrite nanoparticles were generated in two stages: first, metal salts were transformed into hydroxides, and then hydroxides were transformed into nanoferrites. The solid solution of metal hydroxides eventually changed into Zn ferrite by heating at 80 °C, and the subsequent reaction required sufficient time and temperature for this transformation to occur completely; by calcination at a high temperature, one could obtain better crystallinity for the prepared NPs.^61^

**Figure 2:**
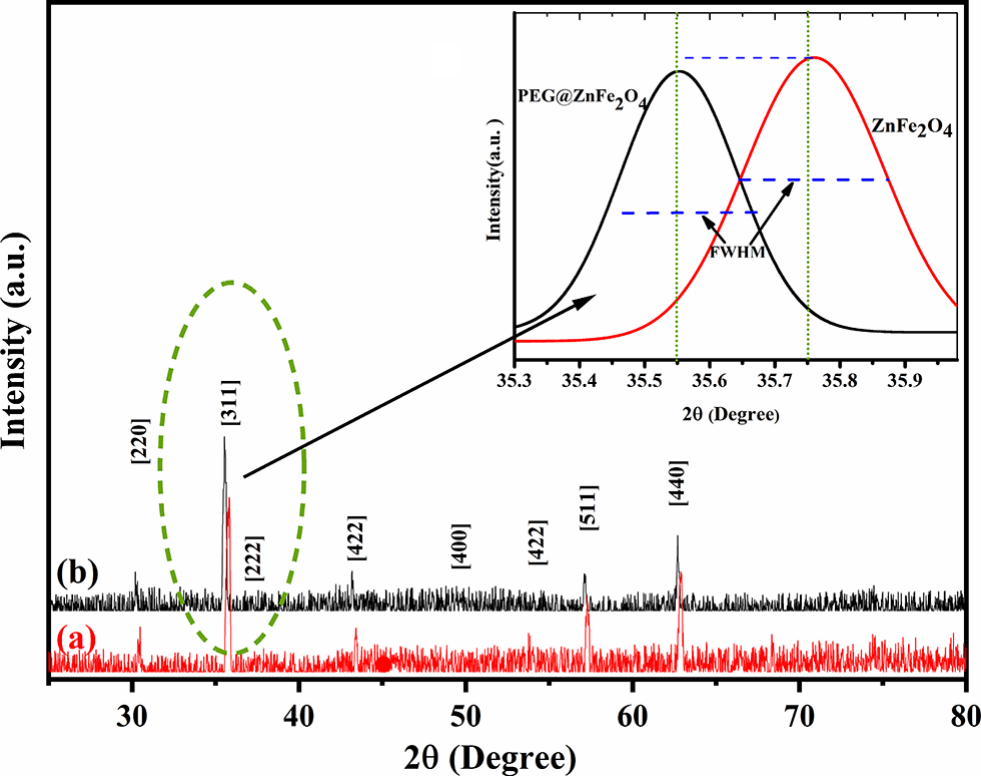
XRD pattern for (a) Zn ferrite NPs and (b) PEG@Zn ferrite NPs with the Gaussian fit of the peaks (311) (inset).

Figure 2 shows the powder XRD pattern for Zn ferrite and PEG@Zn ferrite NPs obtained using the JADE program, with a Gaussian fit of the peaks (311) as an inset. The diffraction peaks associated with Bragg’s reflections of Zn ferrite NPs were observed and successfully indexed as (220), (311), (222), (400), (422), (440) and (511) planes, which correspond to pure phase with spinel structure and match with standard JCPDS card no. 73-1964.^62^ The widening of peaks represents the growth of nanocrystals. The spinel ferrites of PEG@Zn ferrite NPs had diffraction peaks that are comparable to those of Zn ferrite NPs. Similar diffraction patterns of PEG@Zn ferrite NPs suggest that the PEG coating had no influence on the crystalline structure of Zn ferrite NPs.^62^ The obtained data indicate the successful incorporation of the PEG coating on the surface of Zn ferrite NPs.^63^ The XRD pattern (Figure 2) illustrates that after coating with PEG, the crystallite size of Zn ferrite NPs increases. This could be because the interaction of Zn ferrite NPs with polymer (PEG) causes some of the small particles to join together and form large particles, which is also confirmed by the shift in lower angle peak position. A similar behavior has also been reported for nickel zinc ferrite nanoparticles coated with PVA.^64^ Ehi-Eromosele et al. investigated the colloidal stability of Co_0.8_Mg_0.2_Fe_2_O_4_ and demonstrated that, as crystallite size increased after PEG coating, some of the smaller particles may have joined together to form larger particles, which is consistent with the current study.^65^

The structural parameters such as crystallite size, lattice constant (*a*), cell volume (*V*), X-ray density (*d*_*x*_) and hopping lengths (*L*) were calculated using the formulas from the literature.^66^ All calculations use the *d* spacing values and the corresponding (*hkl*) lattice parameters, and Table 1 summarizes the structural parameters for the NPs. As shown in Table 1, the lattice constant characteristics of PEG@Zn ferrite are lower than those of the uncoated samples. The reduction of the average lattice constant is due to the appearance of PEG, which causes the strain value to decrease; such behavior has also been reported by other researchers.^67^

**Table 1:** Structural Parameters Calculated Using X-ray Diffraction Data

**Samples**	***D***_XRD_	***a***	***V***	***L***	***d***_***x***_
Zn ferrite	8	8.3691	586.1951	2.9589	4.23900
PEG@Zn ferrite	14	8.3221	576.3614	2.9423	4.43123

**Figure 3:**
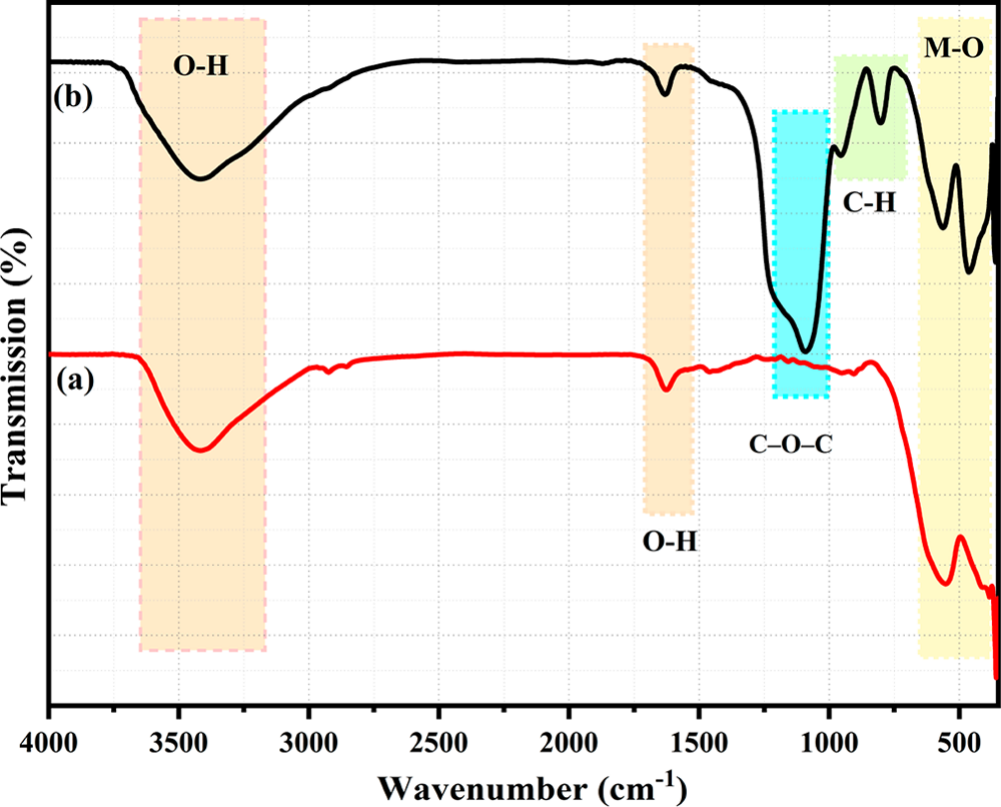
FTIR spectra of (a) PEG@Zn ferrite NPs and (b) Zn ferrite NPs.

FTIR spectroscopy was utilized to identify the bonds responsible for the alteration of NPs by PEG molecules. The absorption spectra of Zn ferrite and PEG@Zn ferrite NPs are depicted in Figure 3. The tetrahedral and octahedral stretching vibrations of metal oxygen emerge as two absorption bands between 300 and 600 cm^–1^, respectively.^66^ The peak observed at 802 cm^–1^ is assigned to the deformation vibration of Fe–OH.^68^ The absorption band at 800–1000 cm^*–*1^ corresponds to the stretching of the C*–*H bonds of PEG. The absorption bands at 3400 and 1600 cm^*–*1^ correspond to the stretching and vibration of the O–H bond, respectively. In addition, the absorption bands at 1086 cm^*–*1^ are the result of the bending vibration of the C–O–C bond.^63^ These results corroborate the coating and adhesion of PEG to the surfaces of the Zn ferrite NPs, and Table 2 provides a description of the bands of Zn ferrite and PEG@Zn ferrite NPs.^29,69,70^

**Table 2:** FTIR Spectra of Zn Ferrite and PEG@Zn Ferrite NPs

**Samples**	**IR region or bands (cm**^**–1**^**)**	**Descriptions**
Zinc ferrite NPs	3418	v(OH) stretching
	1623	v(OH) bending
	403	v(M–O) stretching
	550	v(M–O) stretching
PEG-coated zinc ferrite NPs	3436	v(OH) stretching
	1620	v(OH) bending
	1085	v(C–O–C) stretching
	938	v(C–H) bending
	875	v() bending
	800	v(Fe–OH) stretching
	558	v(M–O) stretching
	443	v(M–O) stretching

**Figure 4:**
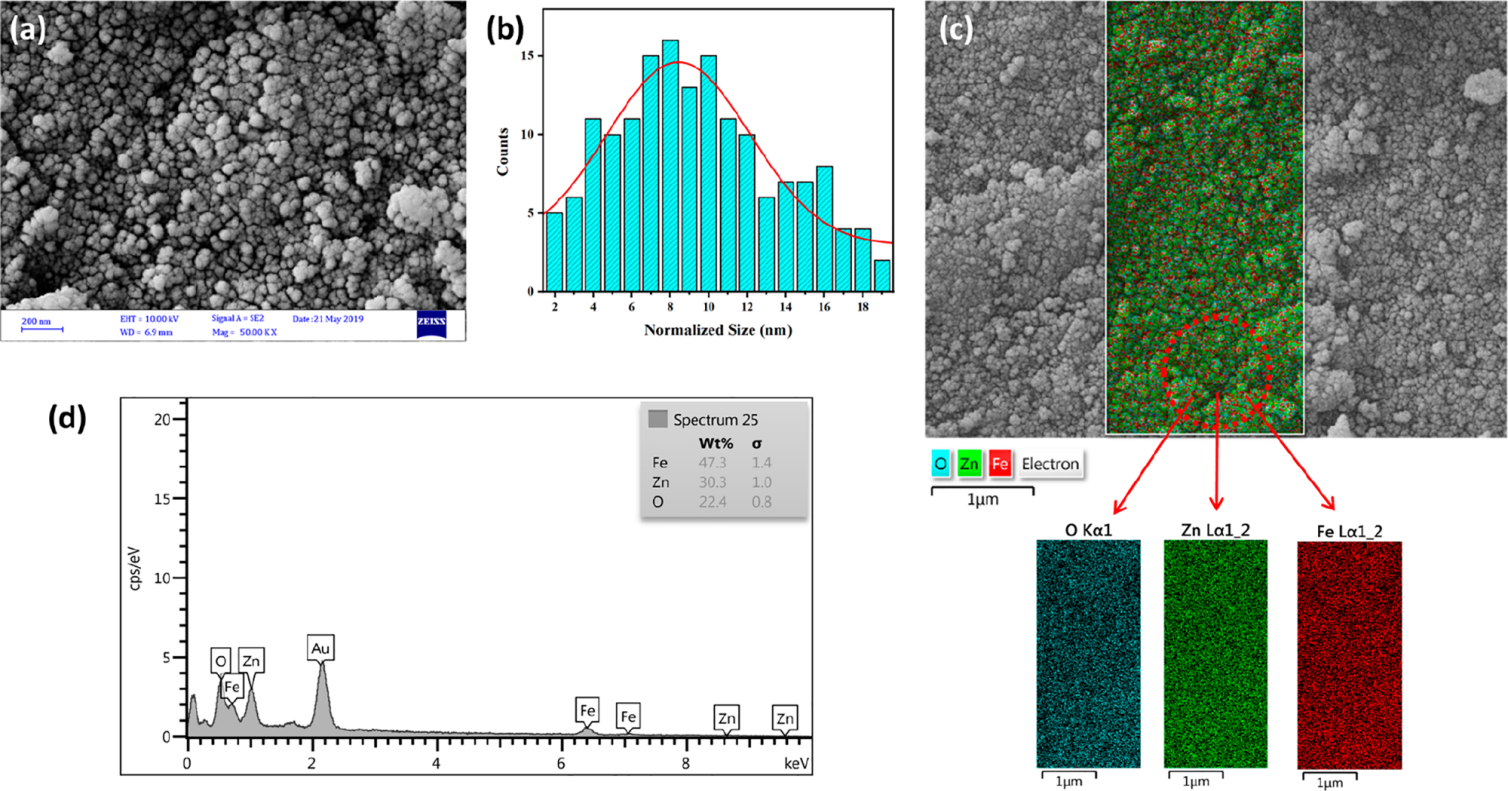
(a) FESEM micrograph, (b) size histogram, (c) EDS profile, (d) EDS mapping of Zn ferrite NPs prepared by hydrothermal method.

The observed FESEM results reveal that the produced Zn ferrite NPs had a spherical form (Figure 4a). The aggregation in the FESEM images is the result of magnetic NP interactions, which have been documented before for many other nanocrystalline spinel ferrites.^71^ As a result of calcination, heat treatment led to agglomeration, which is typical for spinel ferrites. Consequently, it appears unavoidable that agglomeration will occur at the elevated calcination temperature.^71^ Also, the grains appear to have agglomerated in some areas, which might be due to the release of gases during the burning process. The micrograph depicts the formation of powder with an average particle size of less than ∼8–10 nm. This value agrees well with that calculated from the XRD peak broadening. To estimate the average particle size, the diameters of about 160 particles were measured using ImageJ software, and the average particle size histogram is added as Figure 4b.^72^ The related EDS chart of the Zn NPs sample in Figure 4c indicates that Zn, Fe, and O elements can be validated with respective mass percentages of 30.3%, 47.3%, and 22.4% and calculated atomic percentages of 17.03%, 31.11%, and 51.85%, respectively, which are in agreement with the molecular formulation.^73^ It also shows that the calculated mass percentages in Table 3 are comparable with the observed values. As a result, the hydrothermal synthesis process is an effective method for producing zinc ferrite NPs with high homogeneity. The EDS elemental mapping micrographs of Zn ferrite NPs are depicted in Figure 4d. Clearly, the existence of elements Zn, Fe, and O is verified and can be distinguished in the EDS mapping with three distinct colors, indicating that the elemental distributions in the final products are homogeneous and highly pure.

**Table 3:** Elemental Composition of ZnFe_2_O_4_ NPs

**Element**	**Symbol**	**Atomic weight**	**Atoms**	Mass % calculated	Mass % observed	Atomic %
Zinc	Zn	65.38	1	27.12%	30.3%	17.03%
Iron	Fe	55.845	2	46.33%	47.3%	31.11%
Oxygen	O	15.9994	4	26.54%	22.4%	51.85%

**Figure 5:**
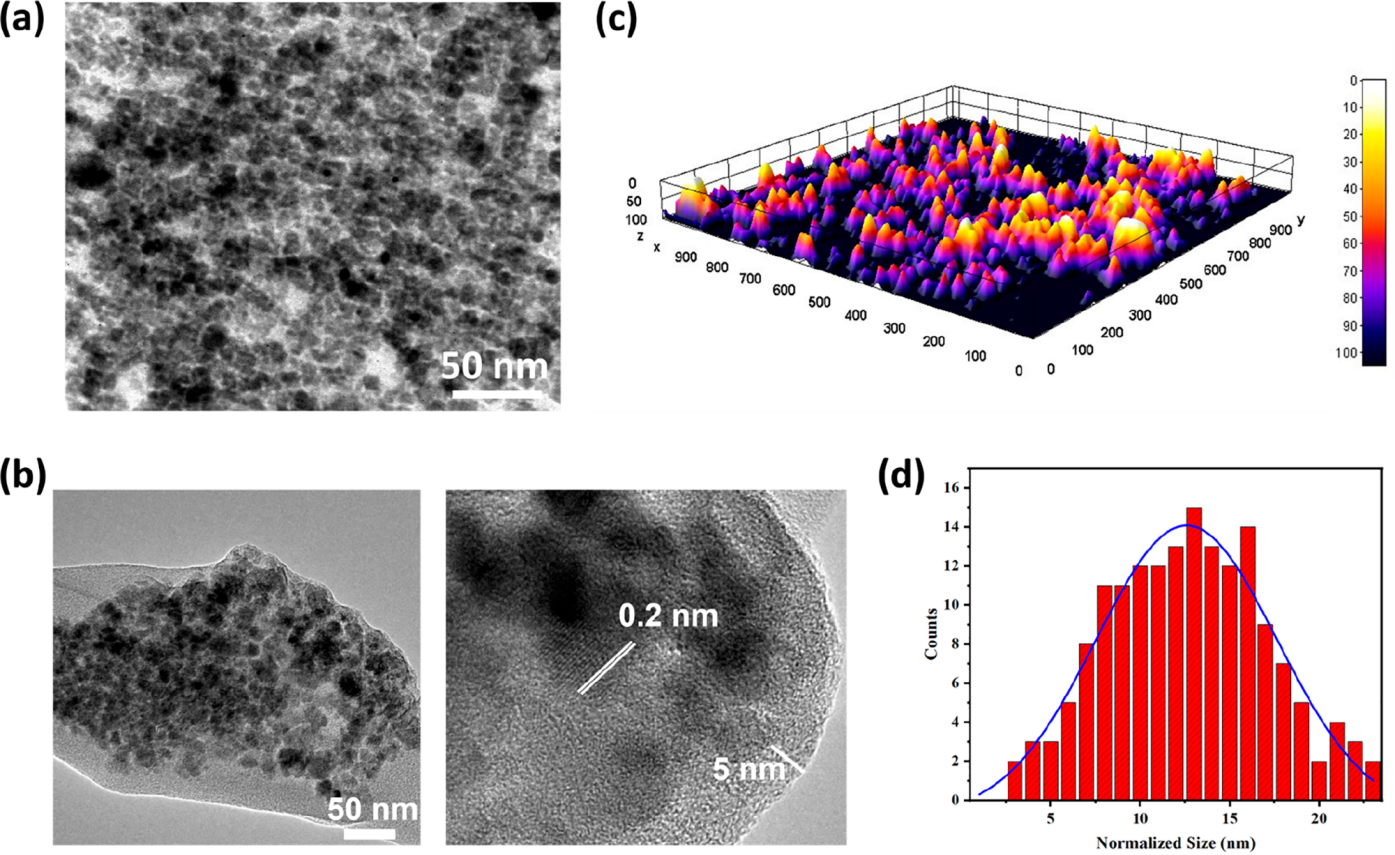
(a) TEM images of uncoated Zn ferrite NPs, (b) HRTEM image of PEG-coated Zn ferrite NPs, (c) 3D structural image of uncoated Zn ferrite NPs and (d) size histogram of PEG-coated Zn ferrite NPs.

TEM investigation revealed that the initial Zn ferrite NPs generated by the hydrothermal technique were less than 10 nm in size (Figure 5a). The particle size distribution was quite homogeneous, and the shape of the produced NPs was mostly spherical. TEM validated the results obtained from FESEM and the crystal size predicted by the Scherrer equation from XRD studies. XRD investigation determined the crystal size to be around 8 nm. Also, it was discovered that uncoated NPs exhibit aggregation. Figure 5b depicts HRTEM images of PEG@ Zn ferrite NPs having a quasi-spherical morphology. The 3D structural images for uncoated NPs confirmed the homogeneous preparation (Figure 5c). After PEG functionalization, the dispersibility of the NPs increased, which could be due to the formation of a nonmagnetic polymer surface layer. These PEG-functionalized NPs had an average diameter of 15 nm, as calculated by the size histogram (Figure 5d), and these biologically active particles display long-term high stability. Lattice fringes with a width of 0.2 nm correspond to the *d* spacing of (311) plane. These differential lattice fringes indicate the crystalline character of the sample formulations. Several small nanoparticles attached together as aggregates by mutual magnetic attractions to form the core and the presence of the PEG layer can be confirmed (Figure 5b).

**Figure 6:**
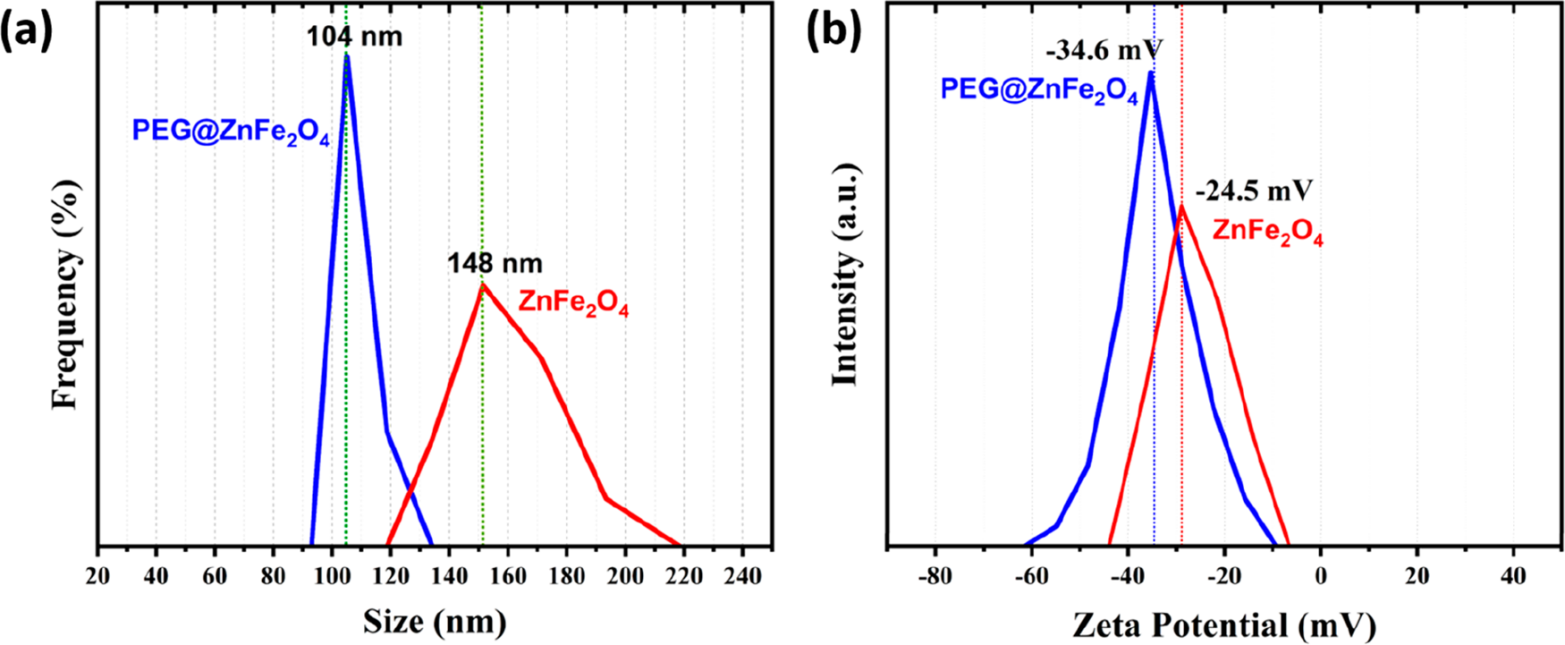
(a) Size distribution and (b) zeta potential measurements of Zn ferrite NPs and PEG@Zn ferrite NPs.

To find out the hydrodynamic sizes and distributions, 0.1 g of suspended Zn ferrite and PEG@Zn ferrite NPs were used. The XRD and morphological results were in good agreement (Table 4). The average hydrodynamic size and distribution of Zn ferrite NPs and PEG@Zn ferrite NPs are shown in Figure 6a and Table 4, respectively. The average size of Zn ferrite NPs was measured in the range of 130–150 nm. Zn ferrite NPs had a larger average particle size than PEG@Zn ferrite NPs (80–105 nm) due to a greater tendency toward agglomeration (Figure 6a). DLS measurements for the coating size were conducted three times, and their averages were taken to give consistent information. The sharp peak corresponds to 148 nm for the uncoated NPs and 104 nm for the PEG-coated NPs, which is the dominant particle size distribution in suspension.

**Table 4:** Zeta Potential, Hydrodynamic Diameter and Polydispersity Index of Zn Ferrite and PEG@Zn Ferrite NPs

**Sample**	**Zeta potential (mV)**	**Mean hydrodynamic diameter (nm)**	**Polydispersity index (PDI)**
Zn ferrite	–24.5	148	0.171
PEG@Zn ferrite	–34.6	104	0.2

Solutes had an average hydrodynamic diameter of 143.72 ± 1.60 nm and a zeta potential of −24.5 mV. The value of the zeta potential suggests that the particles have initial stability in water. Thus, magnetite nanoparticles that are not coated show a modest tendency to aggregate. A polydispersity index of roughly 0.200 implies that the sample is monodisperse. PEG-coating of the Zn ferrite NPs led to a smaller hydrodynamic diameter (103.78 nm), a slightly higher polydispersity index (0.216), and a more stable zeta potential (−34.6 mV) compared to the uncoated Zn ferrite NPs. The stabilizers do not attract each other due to electrostatic repulsion. The negatively charged oxygen atoms in PEG prevent the stacking of polymer layers, resulting in a reduced zeta potential and a more stable colloidal solution. This indicates that the coated sample forms fewer clumps in water than the untreated sample. High charge differences (> ±10 mV) result in increased interparticle repulsion; hence, rising zeta potential values increase colloidal stability. Due to the repetitions of hydrophilic ethylene glycol in the PEG coating, dispersibility in aqueous environments such as water is enhanced.^74^

**Figure 7:**
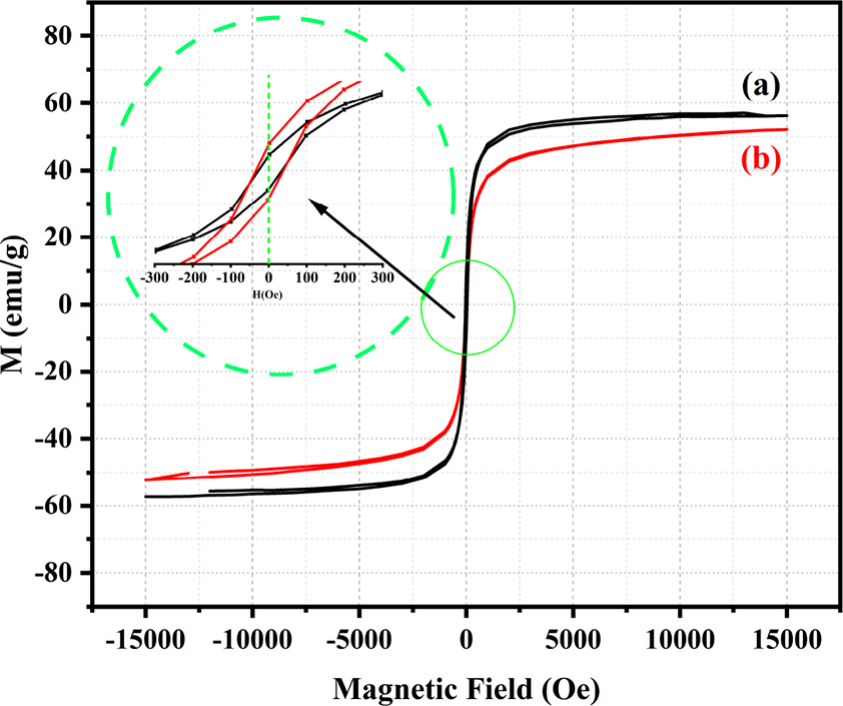
*M*–*H* curves for (a) Zn ferrite NPs and (b) PEG@Zn ferrite NPs.

Figure 7 depicts the *M*–*H* curves of Zn ferrite NPs and PEG-coated Zn ferrite NPs at room temperature in the presence of magnetic fields up to 15000 Oe, obtained by a vibrating sample magnetometer (VSM). Table 5 shows that the saturation magnetization of Zn ferrite NPs was greater than that of PEG-coated Zn ferrite NPs. After coating, the saturation magnetization of Zn ferrite NPs dramatically decreased. Since saturation magnetization is proportional to the mass ratio of the magnetic material within the organic layer, it is hypothesized that after capping each particle, the mass fraction of magnetic material decreases, hence reducing the saturation magnetization.^75^ The magnetic characteristics of Zn ferrite NPs and PEG@Zn ferrite NPs were determined and are presented in Table 5. Both samples exhibited the usual features of superparamagnetic behavior: absence of hysteresis, nearly nonmeasurable coercivity, and remanence. For each of these nanoparticles, these characteristics imply the presence of superparamagnetic and single-domain particles. So, the prepared nanoparticles showed negligible hysteresis and superparamagnetic behavior and a moderate *M*_*s*_ value that tended to achieve enhanced MRI contrast; similar behaviors of ferrite NPs have been reported by other researchers.^51,76,77^ Therefore, the produced nanoparticles displayed reduced coercivity and remanent magnetization, as well as a superparamagnetic behavior, rendering them ideal for biological applications. High saturation magnetization is one of the most prominent aspects in the effective thermal power generation of NPs. Since magnetic measurements indicate that the coated sample is magnetized, the magnetization is proportional to the mass ratio of the magnetic material deposited within the organic layers.^63^ Therefore, the presence of a coating layer over magnetic NPs induces a decrease in saturation magnetization, which influences heat generation.^78^ The percentage of squareness is less than what would be predicted for noninteracting, randomly oriented items, which might be related to the exchange interactions between particles, which influence the magnetic anisotropy of nanopolycrystalline magnetic materials.^66^

**Table 5:** Magnetic Parameters of Zn Ferrite NPs and PEG@Zn Ferrite NPs Obtained from the *M*–*H* Curves

**Samples**	***M***_***s***_ **(emu/g)**	***M***_***r***_ **(emu/g)**	***H***_***c***_ **(Oe)**	***M***_***r***_**/*****M***_***s***_
Zn ferrite NPs	55	13	<100	0.23
PEG@Zn ferrite NPs	51	11	<100	0.21

**Figure 8:**
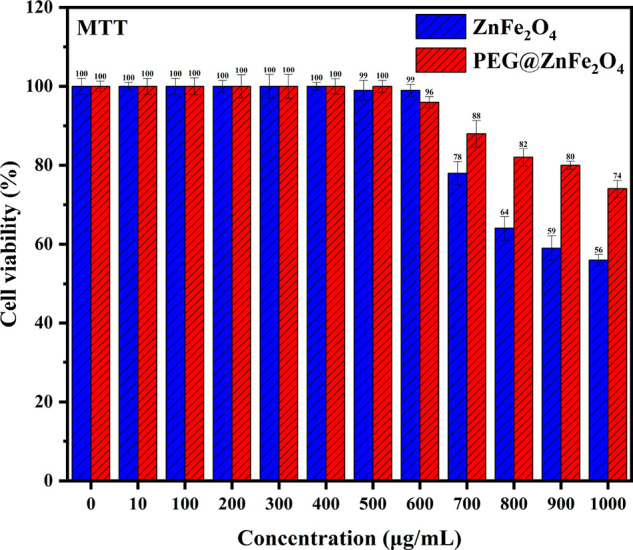
Effect of (a) Zn ferrite NPs and (b) PEG@Zn ferrite NPs on the viability of HSF 1184 cells after incubation for 24 h at different concentrations, as evaluated by MTT assay.

The possible adverse impacts of NP exposure may become a major concern as uses of NPs continue to develop. Understanding the cytotoxicity of NPs to human cell lines is crucial before using them for in vitro or in vivo applications. The fibroblasts have been described as a well-founded resource for in vitro investigations, having considerable benefits over altered cell lines. In addition, they are the most abundant cells in complex organisms. Human normal skin cell lines (HSF 1184) were utilized in this work to examine the cytotoxic potential of the produced PEG@Zn ferrite NPs and Zn ferrite NPs using the MTT test. After 24 h of incubation at several concentrations (0–1000 μg/mL) of Zn ferrite and PEG@Zn ferrite NPs, the percentage of viable cells was determined and is displayed in Figure 8.

Even at 1000 μg/mL concentration, the PEG@Zn ferrite NPs demonstrated no significant cytotoxic impact compared to the control. On the other hand, the cell viability following incubation with Zn ferrite NPs was considerably decreased compared to cells coated with PEG. More than 74% of cell growth was found in the presence of 1000 μg/mL of PEG@Zn ferrite NPs, whereas only 56% of cell survival at the same concentration was reported for Zn ferrite NPs. The results show that as the concentration of Zn ferrite NPs increased above 700 μg/mL, cell proliferation decreased, which could be due to the NPs’ ability to generate reactive oxygen species (ROS). Raised ROS levels can induce mitochondrial malfunction, DNA damage, and protein damage in the cells, leading to the suppression of cell growth and eventually cell death.^79^ All these data clearly revealed that the cytotoxicity of Zn ferrite NPs was dependent on concentration and exposure period, as has been reported in earlier investigations.^80−82^ The Zn ferrite NPs were established to be expressively more cytotoxic to HSF-1184 cells as compared to PEG@Zn ferrite NPs, which might be linked with the inherent anticancer characteristic of Zn ferrite NPs.^83^ In addition, cells treated with PEG@Zn ferrite NPs acquired a considerable viability of approximately 100% at 600 μg/mL concentration and 80% at 900 μg/mL concentration. These results suggest that the biocompatibility of Zn ferrite NPs was significantly enhanced when coated with PEG, as reported elsewhere.^84^

**Figure 9:**
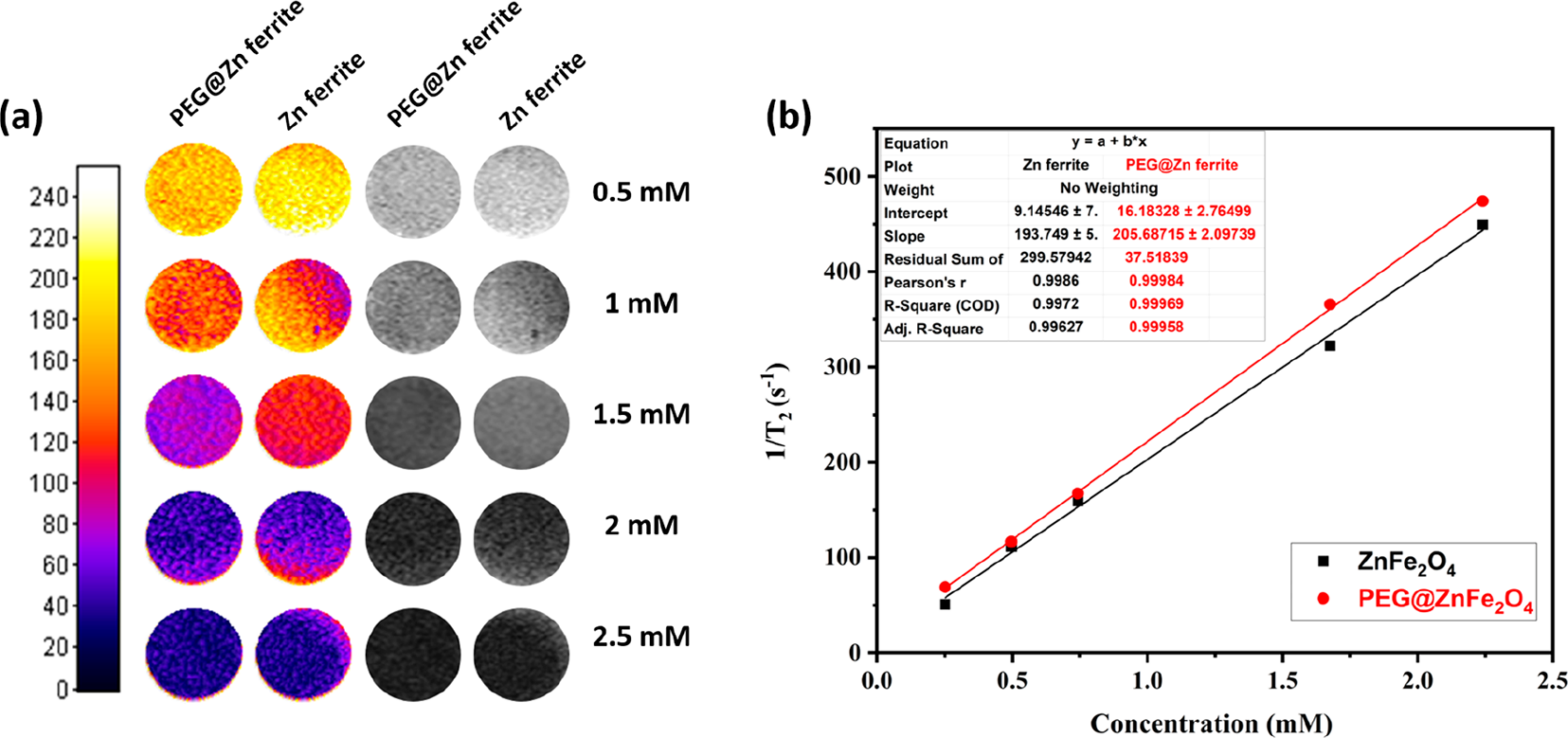
(a) T_2_-weighted MRI images of Zn ferrite and PEG@Zn ferrite NPs at various concentrations. (b) Plot representing the r_2_ relaxation rate versus Zn ferrite and PEG@Zn ferrite NP concentrations that r_2_ values calculated from the slopes of the best-fit lines for the experimental data.

MRI contrast agents can be either positive or negative, depending on the size of the particles, how they are coated, and how magnetic they are. Zn ferrite and PEG@Zn ferrite NPs could be used as a negative contrast agent to measure T_2_ MRI relaxation times in this case. The relativities of Zn ferrite NPs and PEG@Zn ferrite NPs as MRI contrast agents were determined using an agar phantom and a clinical MRI (1.5 T).^27^ When the T_2_-weighted images in the phantom gels were analyzed, increasing NPs concentrations resulted in discernible darkening and negative contrast (Figure 9a). However, T_2_-weighted images for PEG@Zn ferrite NPs were stronger due to better dispersion of the NPs due to the polymer coating. The color map along the T_2_-weighted images displays the various degrees of coated ion concentrations, proving that coated NPs can be a more effective MRI contrast agent.

Figure 9b was derived from the observed values, which were completely linear with increasing Zn ferrite and PEG@Zn ferrite NP concentrations. The slope was estimated using a linear regression line, and it may be related to the form of the NP since the structure has a comparatively lower surface-to-volume ratio, which leads to fewer hydrogen nuclei interactions in the surrounding water.^85^

Zn ferrite NPs had a r_2_ relaxivity of 193 mM^–1^ s^–1^, which is comparable with a previous work that utilized a different synthesis route and structure.^86^ Following the quantum mechanical outer-sphere theory, saturation magnetization has a significant impact on transverse relaxation, and NPs with large *M*_s_ have high r_2_ values.^87,88^ In agreement with this, PEG@Zn ferrite clusters had a greater r_2_ of 205 mM^–1^ s^–1^. Compared to uncoated nanoparticles, the r_2_ relaxivity was considerably increased, and it is thought that the amplification of spin–spin relaxation reflects the capacity of magnetic particles to deform the local magnetic field. Some sources claim categorically that size is the decisive factor. For instance, when the particle size is greater than 9 nm and the *M*_*s*_ value is high, the r_2_ relaxivity will increase due to the aggregation in the suspension. The results show that PEG@Zn ferrite NPs might be employed as a superior T_2_-shortening agent because of their tiny size and large r_2_ value. In the T_2_-weighted MR images, the sample with a short T_2_ relaxation time (that is, a high r_2_ value) shows a dark signal. These samples’ T_2_-weighted phantom imaging confirms the trend of their T_2_ contrast abilities.

## Conclusion

The results indicate that the modification technique affected the final morphology, size, agglomeration degree, and charge of the nanoparticles. PEG-coated zinc ferrite NPs synthesized by the hydrothermal process with ultrasmall size (15 nm), good and homogeneous dispersion (biocompatible and water dispersible), and spherical shape could be extremely desirable for biomedical and clinical applications. FTIR and TEM studies confirmed the coating of the biocompatible PEG polymer. The zeta potential value demonstrated that the coated NPs are more stable than the uncoated NPs, while DLS analysis determined the dispersibility and liquid stability of NPs as a viable choice for biomedical applications. To verify the biocompatibility of the created nanosystem, an in vitro MTT experiment was performed. The cytotoxicity experiments conducted on HSF 1184 cell lines revealed that NPs exhibited a substantial dosage advantage over uncoated NPs, and the coated synthetic NPs were nontoxic, making them an excellent contender for biomedical applications. The PEG@Zn ferrite NPs demonstrate significant potential in MRI, and the current production and assessment procedures for the NPs might be applied to produce other types of magnetic NPs. Furthermore, in terms of NP concentration in aqueous solutions, PEG@Zn ferrite NPs had a high r_2_ relaxivity (205 mM^–1^ s^–1^). Further studies should be conducted to determine whether ferrite-based NPs of other shapes or coating materials may also be used as T_2_ contrast agents.

## Data Availability

The authors confirm the absence of sharing data.
